# Comparative analysis of the ability of *Clostridium clariflavum* strains and *Clostridium thermocellum* to utilize hemicellulose and unpretreated plant material

**DOI:** 10.1186/s13068-014-0136-4

**Published:** 2014-11-18

**Authors:** Javier A Izquierdo, Sivakumar Pattathil, Anna Guseva, Michael G Hahn, Lee R Lynd

**Affiliations:** Thayer School of Engineering, Dartmouth College, Hanover, NH USA; BioEnergy Science Center Oak Ridge National Laboratory Oak Ridge, Oak Ridge, TN USA; Department of Biology, Hofstra University, Hempstead, NY USA; Complex Carbohydrate Research Center, University of Georgia, Athens, GA USA

**Keywords:** CBP, *Clostridium thermocellum*, *Clostridium clariflavum*, Hemicellulose, Switchgrass

## Abstract

**Background:**

Among themophilic consolidated bioprocessing (CBP) candidate organisms, environmental isolates of *Clostridium clariflavum* have demonstrated the ability to grow on xylan, and the genome of *C. clariflavum* DSM 19732 has revealed a number of mechanisms that foster solubilization of hemicellulose that are distinctive relative to the model cellulolytic thermophile *Clostridium thermocellum*.

**Results:**

Growth experiments on xylan, xylooligosaccharides, and xylose reveal that *C. clariflavum* strains are able to completely break down xylan to xylose and that the environmental strain *C. clariflavum* sp. 4-2a is able to grow on monomeric xylose. *C. clariflavum* strains were able to utilize a larger proportion of unpretreated switchgrass, and solubilize a higher proportion of glucan, xylan, and arabinan, with strain 4-2a reaching the highest extent of solubilization of these components (64.7 to 69.4%) compared to *C. thermocellum* (29.5 to 42.5%). In addition, glycome immunoanalyses of residual plant biomass reveal differences in the extent of degradation of easily accessible xylans, with *C. clariflavum* strains having increased solubilization of this fraction of xylans relative to *C. thermocellum*.

**Conclusions:**

*C. clariflavum* strains exhibit higher activity than *C. thermocellum* in the breakdown of hemicellulose and are capable of degrading xylan to xylooligomers and xylose. This capability seems to also play a role in the higher levels of utilization of unpretreated plant material.

**Electronic supplementary material:**

The online version of this article (doi:10.1186/s13068-014-0136-4) contains supplementary material, which is available to authorized users.

## Background

Consolidated bioprocessing, or CBP, is one of the most promising and widely recognized strategies for the microbial conversion of lignocellulosic biomass into products of interest [1], with thermophilic, cellulolytic bacteria often considered in this context. It is curious to note, however, that the model thermophile *Clostridium thermocellum* and several of its close relatives do not grow on hemicellulose or its component pentose sugars. Indeed, the only moderately thermophilic (optimal growth temperature about 60°C) cellulolytic anaerobes we know of that grow on xylose, xylooligomers, or other hemicellulose components are an environmental isolate of *C. clariflavum* previously reported by our group [2] and a distant relative of these clostridia, *C. stercorarium* [3]. Other clostridia from the same phylogenetic cluster (Cluster III), such as *C. cellulolyticum*, can utilize both cellulose and hemicellulose, but are able to do so at mesophilic temperatures, which results in slower fermentation rates [4].

The type strain of *Clostridum clariflavum*, DSM 19732, was originally isolated from anaerobic sludge and is a very close relative of fellow cellulolytic thermophiles within Clostridia Cluster III, *C. thermocellum* and *C. straminisolvens* [5,6]. Much like *C. thermocellum*, this organism employs a cellulosome to break down plant biomass and can grow very effectively on cellulose but not on hemicellulose. In an effort to expand the known metabolic diversity of this species, we have previously described the enrichment and isolation of novel *C. clariflavum* strains from thermophilic compost, and reported on their ability to grow on xylan, which is a key differentiating capability compared to DSM 19732 [2,7]. In addition, the genome of *C. clariflavum* DSM 19732 reveals a very distinct mechanism for the breakdown of hemicellulose, as compared to its close relative *C. thermocellum* [8].

In the work reported here, we explore and characterize the ability of two representative *C. clariflavum* strains, the type strain DSM 19732 and environmental isolate strain 4-2a, to utilize hemicellulose, both in the form of C5 monomers and polymers (xylose, xylooligosaccharides, xylan) and in unpretreated plant material, and compare these characteristics to those of *C. thermocellum*.

## Results

### Dynamics of xylan utilization

**Figure 1:**
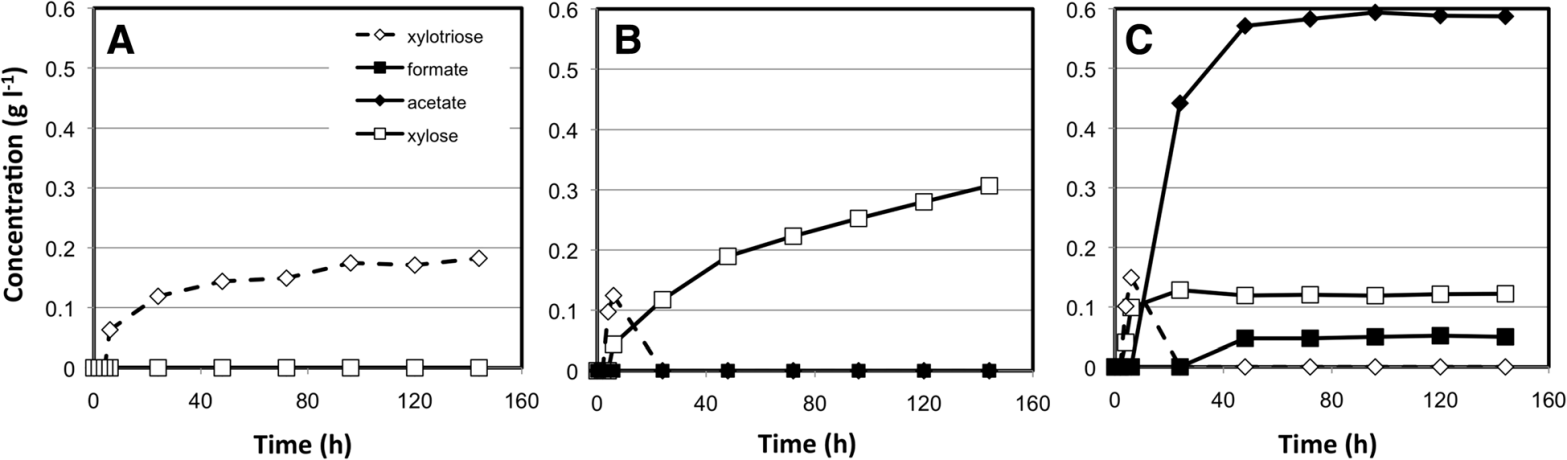
**Dynamics of soluble product formation from xylan growth experiments.** The dynamics of products of xylan hydrolysis (xylotriose and xylose) and fermentation products (acetate, formate) are shown for growth experiments for *C. thermocellum* ATCC 27405 **(A)**, *C. clariflavum* DSM 19732 **(B)**, and *C. clariflavum* str. 4-2a **(C)**, using xylan as the only carbon source.

The dynamics of xylan hydrolysis were examined for *C. clariflavium* strains DSM 19732 and 4-2a as well as *C. thermocellum.* All three strains exhibited different xylan hydrolysis and utilization behaviors (Figure 1). *C. thermocellum* inocula were able to hydrolyze xylan to xylooligomers (such as the xylotriose detected in this experiment), but were not able to grow on xylan or produce any detectable xylose (Figure 1A). *C. clariflavum* DSM 19732 was able to release xylooligosaccharides from the xylan substrate that were quickly broken down to xylose, but was also not able to grow on these substrates (Figure 1B). Hydrolysis of xylan and xylooligosaccharides to xylose slowly continued beyond 144 hours, resulting in a final measurement of 0.3 g xylose/L. Both *C. thermocellum* and *C. clariflavum* DSM 19732 experiments provide evidence of enzymatic breakdown of xylan by the inocula utilized, but no growth. *C. clariflavum* str. 4-2a was able to grow on 3 g/L xylan (Figure 1C). Xylooligosaccharides (in the form of xylotriose) were observed early in the fermentation process, but appear to be quickly hydrolyzed all the way to xylose. Some residual xylose was not utilized by *C. clariflavum* str. 4-2A and accumulates to a final concentration of 0.12 g/L. In addition to turbidity and microscopic examination indicative of cell growth, formation of acetic acid and formic acid by str. 4-2A, but not other strains, provided evidence of fermentation.

### Dynamics of xylooligosaccharide utilization

**Figure 2:**
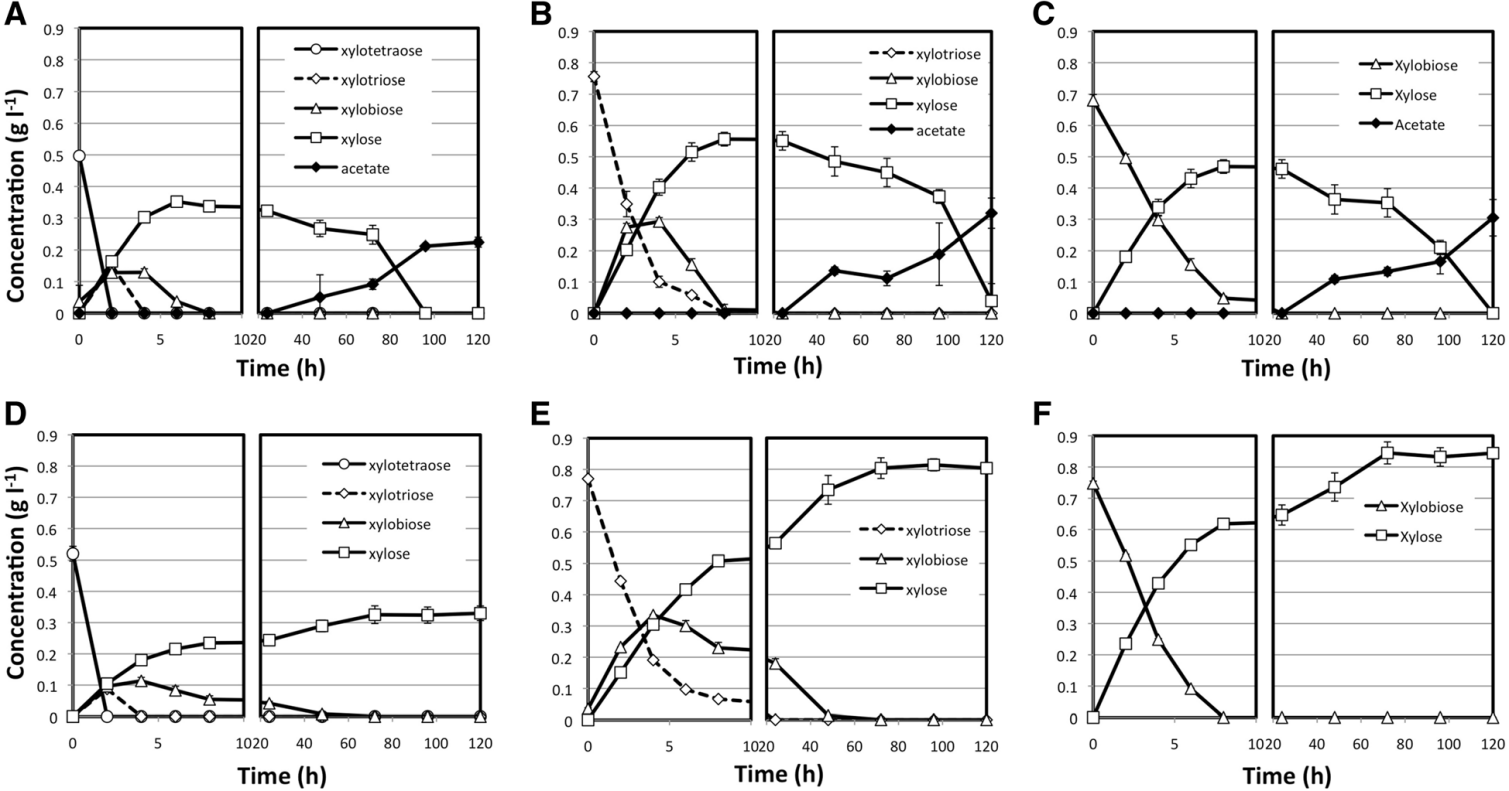
**Dynamics of xylooligosaccharide utilization for** ***C. clariflavum*** **strains.** The breakdown dynamics and fermentation product formation are shown for triplicate experiments using xylotetraose **(A**, **D)**, xylotriose **(B**, **E)**, and xylobiose **(C**, **F)**, for growth experiments inoculated with *C. clariflavum* str. 4-2a **(A**, **B**, **C)** and *C. clariflavum* DSM 19732 **(D**, **E**, **F)**.

*C. clariflavum* strain 4-2a was able to grow on all three xylooligosaccharides (xylobiose, xylotriose, and xylotetraose) we tested (Figure 2), with similar behavior as observed for xylan. For all three xylooligosaccharides, larger molecules were completely broken down into smaller components within the first 24 hours of growth. Xylose that resulted from xylooligosaccharide hydrolysis was slowly fermented over 100 to 120 hours, but eventually was completely utilized (Figure 2A, B, C). Acetic acid production was detected at the same time as xylose utilization was observed. In comparison, *C. clariflavum* strain DSM 19732 was able to break down all xylooligosaccharides to xylooligosaccharides of smaller size and eventually all the way to xylose, but it was not able to utilize the resulting xylose, which resulted in accumulation of xylose in growth experiments using all three xylooligosaccharides (Figure 2D, E, F). The lack of fermentation products (acetate) further confirms the inability of this organism to utilize xylose resulting from xylooligosaccharide hydrolysis. No growth was observed in *C. thermocellum* ATCC 27405 cultures growing on the three xylooligosaccharides tested.

### Dynamics of xylose utilization

**Figure 3:**
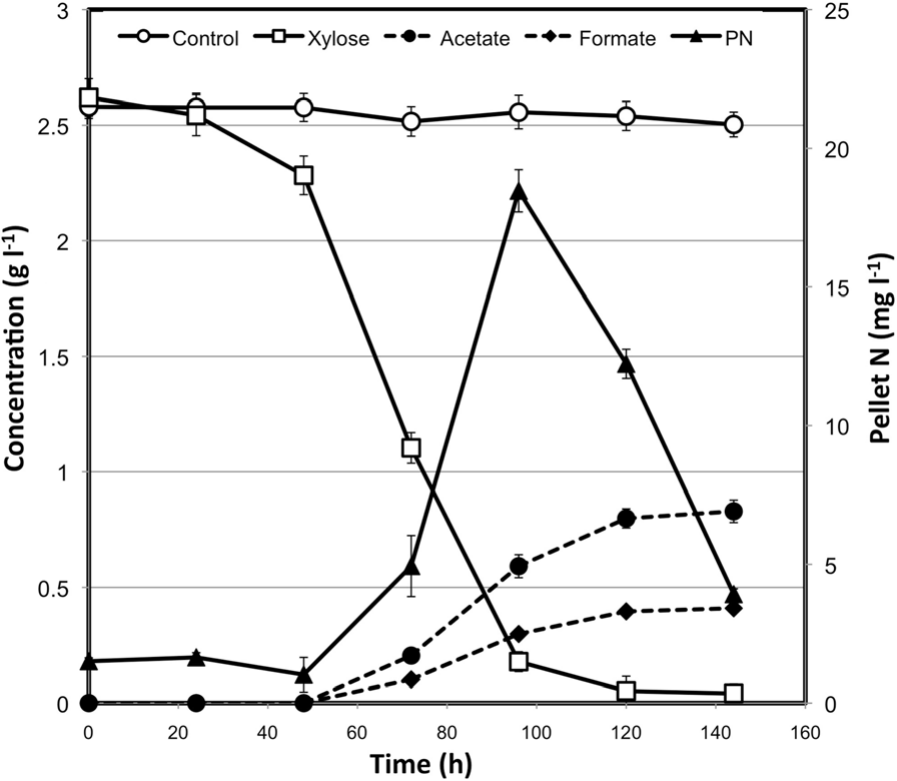
**Xylose utilization by** ***C. clariflavum*** **strain 4-2a.** Xylose utilization and fermentation product dynamics are shown for triplicate growth experiments. The dynamics of pellet nitrogen accumulation, as a proxy for cell growth, is also shown. Control values represent the concentration of xylose after inoculation with *C. clariflavum* DSM 19732.

*C. clariflavum* strain 4-2a was able to grow on xylose as the sole carbon source (Figure 3). Evidence of fermentation was provided by the detection of acetate and formate between 48 and 72 hours after inoculation. Likewise, evidence of cell growth was provided by the observed increased cell mass and total pellet nitrogen content over the first 96 hours of culture, which was followed by a decline thereafter. Complete utilization of 2.5 g/L of xylose was slow, taking approximately 120 hours. However, and by comparison, *C. thermocellum* ATCC 27405 and *C. clariflavum* DSM 19732 were not able to grow on xylose, as demonstrated by the lack of xylose utilization in the control experiment with *C. clariflavum* DSM 19732. Similar behavior was observed in bottle experiments with *C. thermocellum* ATCC 27405 (data not shown).

### Conversion of unpretreated switchgrass

**Figure 4:**
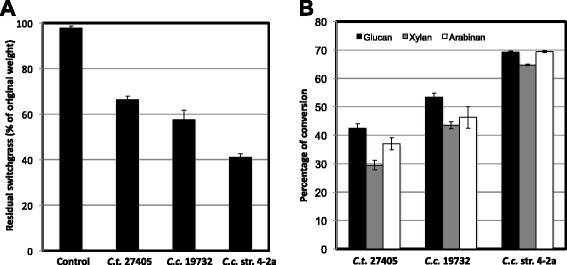
**Unpretreated switchgrass utilization by** ***C. thermocellum*** **and** ***C. clariflavum*** **strains.** Percentage of residual switchgrass relative to original weight **(A)** is shown for triplicate experiments with *C. thermocellum* ATCC 27405, *C. clariflavum* DSM 19732, *C. clariflavum* str. 4-2a, and uninoculated controls after 5 days of incubation. Percentage of glucan, xylan, and arabinan utilization **(B)** is also shown for all three organisms.

After 5 days of incubation on switchgrass without pretreatment beyond autoclaving, batch fermentations with *C. clariflavum* str. 4-2a contained residual plant material averaging 41.2 ± 1.4% relative to the weight of the original plant material (58.8% solubilization). *C. clariflavum* DSM 19732 and *C. thermocellum* ATCC 27405 fermentations contained 57.6 ± 4.1% (42.4% conversion) and 66.5 ± 1.5% (32.5% conversion), respectively (Figure 4A). A set of uninoculated controls displayed little weight loss after incubation at 55°C, with 97.9 ± 0.8% of the original plant material being accounted for. Similarly, residual glucan, xylan, and arabinan contents measured by quantitative saccharification in all of these experiments were higher after inoculation with *C. thermocellum* than with *C. clariflavum* strains (Figure 4B). Quantitative saccharification to determine the sugar composition of uninoculated switchgrass controls resulted in a baseline composition of 35.5 ± 0.7% glucan, 24.5 ± 0.6% xylan, and 3.5 ± 0.2% arabinan. Conversion percentages were calculated using these values and the residual glucan, xylan, and arabinan measured in the inoculated experiments. As seen in Figure 4B, *C. clariflavum* str. 4-2a was the most efficient of the three strains in terms of conversion of switchgrass components, reaching between 64.7% (xylan) and 69.4% (arabinan) conversion. Also, *C. clariflavum* DSM 19732 is more efficient than *C. thermocellum* ATCC 27405, breaking down all three components with conversion percentages in the 43.5 to 53.5% range compared to 29.5 to 42.5% in *C. thermocellum* (Figure 4B).

### Switchgrass glycomics

**Figure 5:**
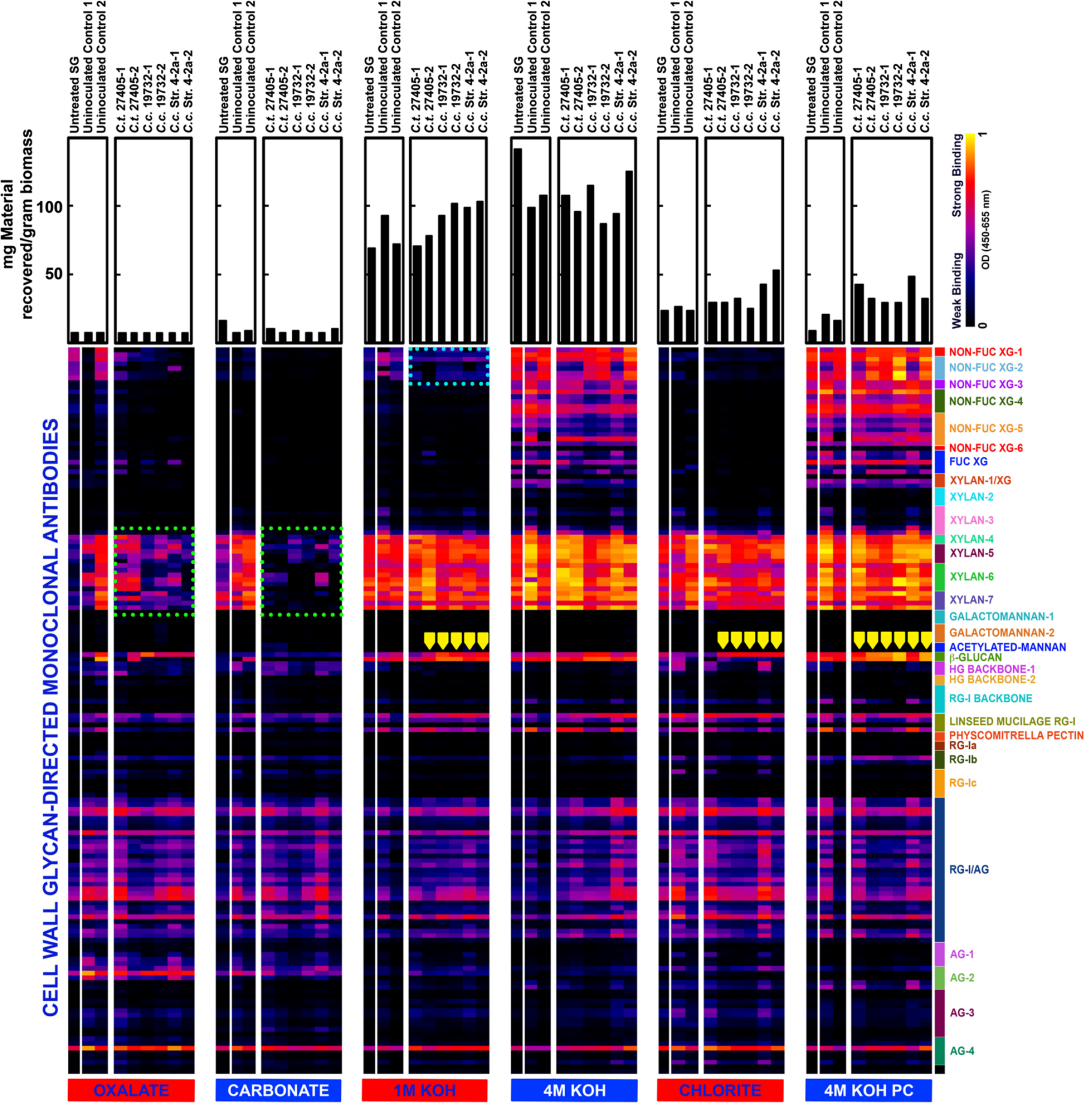
**Glycome profiling of untreated, uninoculated, and microbial incubated switchgrass biomass residues, shown for two biological replicates.** Sequential cell wall extracts were prepared from switchgrass biomass samples using increasingly harsh reagents as explained in the Methods section. The ELISA response values are represented as heatmaps with bright yellow-red-dark blue scale indicating the intensities of the ELISA signals (bright yellow, red, and dark blue colors depict strong, medium, and no binding, respectively). The monoclonal antibodies are grouped based on the cell wall glycans they detect as depicted in the panel at the right-hand side of the heatmap. The gravimetric amounts of carbohydrate materials extracted from the walls by each extraction reagent are shown as bar graphs at the top of the figure.

To obtain further insights into cell wall glycan utilization during microbial growth on switchgrass biomass, glycome profiling analyses were conducted on untreated switchgrass (original plant material), uninoculated switchgrass controls (original plant material incubated in media without bacteria), and switchgrass incubated with microbial strains, C*. thermocellum* ATCC 27405, *C. clariflavum* DSM 19732, and *C. clariflavum* str. 4-2a (Figure 5). First, two notable differences were observed in the glycome profiles of uninoculated controls compared to those of untreated switchgrass. The most notable such difference was the significantly higher abundance of xylan epitopes recognized by the xylan-4 through −7 groups of monoclonal antibodies (mAbs) in the oxalate and carbonate extracts obtained from uninoculated controls compared to those of untreated residues. In addition, there was an increased abundance of pectic-arabinogalactan epitopes in nearly all extracts isolated from uninoculated controls in comparison to untreated switchgrass. Interestingly, the glycome profiling analyses revealed distinct changes in overall cell wall glycan composition and extractability in switchgrass residues that had been incubated with any of the microbial strains in comparison to those of uninoculated controls. All such differences are indicated with green and blue squares in Figure 5. In general, an overall reduction in the abundance of xylan epitopes (as indicated by the reduced binding intensities of xylan-4, xylan-5, xylan-6, and xylan-7 groups of mAbs) was apparent in the oxalate and carbonate extracts isolated from switchgrass residues that had been incubated with any of the three microbial strains in comparison to those of uninoculated controls. Indeed, almost all of the carbonate extractable xylan that was immunologically detectable was absent in the microbially digested wall residues. However, in the oxalate extracts, both *C. clariflavum* strains exhibited increased depletion of xylan epitopes (as indicated by the reduced intensities of mAb binding) compared to that of the *C. thermocellum* strain. In general, the glycome profiles of microbe-incubated biomass did not exhibit any significant variations in the case of the other alkaline and chlorite extracts (harsh extracts). Some subtle differences were observed among extracts isolated from microbe-incubated biomass, such as a depletion of 1 M KOH-released xyloglucan epitopes and a marginal reduction in some 4 M KOH-released xyloglucan epitopes, and a small increase in the abundance of β-glucan epitopes in 1 M KOH and 4 M KOH PC extracts. Overall, the glycome profiling studies reveal a preferential utilization of loosely associated hemicellulosic xylans in switchgrass biomass by all three microbial strains studied, with *C. clariflavum* strains being more efficient in terms of xylan solubilization.

## Discussion

In this work we have tested and demonstrated the ability of *C. clariflavum* strains to utilize hemicellulose as it relates to the model organism and close relative, *C. thermocellum*. At various levels of complexity (xylose, xylooligosaccharides, xylan, and unpretreated plant material), hemicellulose breakdown was markedly different between *C. thermocellum* and *C. clariflavum* strains. Compared to *C. thermocellum,* our results indicate that *C. clariflavum* strains have a distinctive ability to break down xylan into smaller xylooligosaccharides all the way to xylose within a very short amount of time. Our results also make it evident that this is an inherent enzymatic capability of *C. clariflavum* strains, regardless of whether they are able to grow on xylose or not.

A key differentiating factor for *C. clariflavum* strain 4-2a relative to the two other strains examined is its ability to utilize xylose for growth. In all experiments using xylan, xylooligosaccharides, and xylose as sole carbon sources, it was possible to confirm the partial to full utilization of the 5-carbon monomer, although at a much slower rate than the rate of oligomer solubilization. Also, comparatively, *C. clariflavum* strains DSM 19732 and 4-2a grow much faster on cellobiose than the growth displayed by strain 4-2a on xylose. Xylose isomerase and xylulose kinase are both essential genes for the utilization of xylose in Clostridia [9]. Neither gene is found in *C. thermocellum*, and only xylulose kinase is present in *C. clariflavum* DSM 19732 [8]. The genome of strain 4-2a has recently been completed, and we have been able to find a gene for xylose isomerase to be present in this strain, along with the xylulose kinase gene already detected in DSM 19732. This largely explains our observations of xylose utilization by strain 4-2a, as compared to DSM 19732 and *C. thermocellum*. There are also key differences in the ß-xylosidases found in *C. thermocellum* versus those found in *C. clariflavum*. For example, the GH39 ß-xylosidase present in *C. clariflavum* is closely related to similar enzymes in xylanolytic Clostridia that include *C. stercorarium* BxlB, an enzyme demonstrated to break down xylooligomers to xylose [10], and a xylosidase found in *Clostridium* sp. DL-VIII [11], whereas the one found in *C. thermocellum* has sequence homology to xylosidases found in more distantly related actinomycetes. Similarly, most of the GH43 ß-xylosidases found in the *C. clariflavum* genome have no homologs among the fewer representatives found in the *C. thermocellum* genome.

An increasing number of organisms have been successfully discovered and developed to utilize both cellulose and hemicellulose polymers and sugars [12-14]. However, one limitation identified in many of these cases has been the need to use yeast extract as part of the growth medium for these organisms to be able to grow on these complex substrates [15,16]. We have observed, in previous work [2] and in the work reported in this paper, that one feature of *C. clariflavum* species is their demonstrated capability to grow on cellulose and hemicellulose components of plant biomass in defined media without the addition of yeast extract. This should represent a beneficial feature of this organism if it is to be developed for larger scale processes.

*C. clariflavum* strains demonstrated a higher extent of conversion of unpretreated switchgrass than did *C. thermocellum*, both in terms of total conversion (net residual weight) and the ability to break down polymers containing glucan, xylan, and arabinan, with strain 4-2a exhibiting the highest conversion of the three strains examined. In terms of net residual weight, all three clostridial species seem to utilize unpretreated switchgrass extensively (33 to 59% solubilization). Similarly, extreme thermophiles that are also able to utilize both cellulose and hemicellulose, such as *Caldicellulosiruptor bescii,* have been reported to achieve 20 to 25% conversion of unpretreated switchgrass [14,16]. *C. bescii* has been shown to further utilize material after successive reinoculations and fermentations, which was not part of our study with the three Clostridia discussed in this paper.

Glycome profiling of the utilized switchgrass also revealed higher efficiency in breaking down various xylan fractions by *C. clariflavum* strains as compared to *C. thermocellum. C. clariflavum* DSM 19732 possesses a broader array of hemicellulose-active enzymes compared to its close relative *C. thermocellum* as reported in its genome sequence [8], and preliminary exploration of the genome of strain 4-2a has revealed similar findings. Given that the rates of cellulose utilization in *C. thermocellum* and *C. clariflavum* strains are very similar (data not shown), it is possible to hypothesize that the expanded ability of *C. clariflavum* strains to break down hemicellulose may play a role in reducing inhibition of cellulases due to the presence of xylans and xylooligosaccharides [17,18], which may potentially explain the increased ability of both *C. clariflavum* strains to break down the cellulose fraction in unpretreated switchgrass. However, in the case of all three strains, the breakdown of xylan only happens in the loosely associated layers (as detected in oxalate and carbonate fractions of glycome profiling) and not necessarily in the more difficult residual fractions, which likely accounts for the residual glucan, xylan, and arabinan detected through quantitative saccharification. Similar fractional solubilization of cellulose and hemicellulose was observed for all three Clostridia examined here, and has also been observed for *C. bescii* growing on unpretreated switchgrass [16]. Likewise, considering that these experiments were performed with unpretreated switchgrass, any kind of chemical pretreatment that enables access to these more recalcitrant residual fractions will help to overcome this limitation.

It should be noted that *C. clariflavum* and *C. thermocellum* both utilize a cellulosome for the breakdown of plant biomass [8,19]. Although cellulosomes provide a multitude of benefits for Clostridia growing on solid substrates, purified cellulosomes of *C. thermocellum* have been observed to have very low conversion rates in unpretreated switchgrass, as compared to uncomplexed enzymes [20]. Resch *et al.* [20] found that only 20% of glucan from unpretreated switchgrass was broken down by *C. thermocellum* cellulosomes, while we detect 42.5% glucan conversion with *C. thermocellum* and up to 68.7% in *C. clariflavum* str. 4-2a. This discrepancy is possibly due to the fact that, under standard culture conditions, cellulosomal organisms also benefit from a large number of free cellulases, untethered cellulosomal structures, and tethered cellulosomes [8,21], as well as the continuous removal and utilization of released sugars.

## Conclusions

In summary, the present study provides ample evidence of how *C. clariflavum* strains exhibit higher extents of hemicelluose breakdown than *C. thermocellum* given their ability to degrade xylan to xylooligomers and xylose, and, in the case of strain 4-2a, their ability to grow on xylose. This capability may also play a role in the higher levels of utilization of unpretreated plant material, as evidenced in total residual xylan composition and the fractions of easily accessible xylan utilized by these strains when growing on unpretreated switchgrass, as revealed by glycome profiling. Further exploration of other unique metabolic capabilities of *C. clariflavum* strains should be of great interest in the exploration and development of new CBP candidate organisms.

## Methods

### Strains, media, and growth conditions

Type cultures of *C. clariflavum* DSM 19732 and *Clostridium thermocellum* ATCC 27405 were obtained from DSM and ATCC, respectively. *Clostridium clariflavum* str. 4-2a was previously isolated from thermophilic compost enrichment cultures [2]. All growth experiments were conducted using defined MTC media, prepared as previously described [22]. For experiments using xylooligosaccharides as the sole carbon source, all experiments were conducted in serum tubes using small concentrations (0.5 to 0.8 g/L) of xylobiose, xylotriose, and xylotetraose (Megazyme, Bray, Ireland) with a total volume of 12 mL per experimental replicate. All other experiments were conducted in serum bottles with a total volume of 50 mL per experimental replicate. In experiments using xylose (Sigma) as the carbon source, substrate was added after filter sterilization to the other components of MTC. For experiments using solid substrates as the sole carbon source, triplicate serum bottles were prepared using either 3 g/L birchwood xylan (Sigma) or switchgrass. Unpretreated switchgrass (Alamo, sieved 20/80 mesh fraction, grown in Athens, GA, harvested April 2011) was obtained from Dr. Ajaya Kumar Biswal and Dr. Debra Mohnen, University of Georgia, GA. Prior to its use in the growth experiments, the switchgrass was washed in consecutive washes with shaking until no soluble sugars were detected in the supernatant. Triplicate uninoculated control experiments were set up without any organisms and incubated at the same growth temperature as the experiments with inocula.

### Batch cultivation in fermentors

For the switchgrass glycomics analyses, experiments were conducted in larger volumes in 1-liter Biostat Qplus fermentors (Sartorius Stedim, Gottingen, Germany), with a working volume of 1 liter and without pH control. The fermentors were equipped with Norprene tubing (Cole Palmer Instrument Company, Vernon Hills, IL) to minimize oxygen diffusion. Cultures were grown in the same MTC medium used for batch studies, with 3 g of switchgrass and with reducing agents and vitamins added separately after the autoclaving of fermentor vessels. Cultures were stirred at 100 rpm and were sparged with 100 mL min^−1^ nitrogen gas. Each fermentation experiment was performed in separate duplicate fermentations and with a 5% (v/v) inoculum of either *C. thermocellum* ATCC 27405, *C. clariflavum* DSM 19732, or *C. clariflavum* str. 4-2a. In addition, duplicate uninoculated reactors were run with switchgrass for the duration of the same inoculated experiments as uninoculated switchgrass controls. At the end of each 5-day fermentation period, residual plant material was washed, dried, weighed, and stored at −80°C prior to the glycomics analyses.

### Analytical methods

The concentrations of sugars and fermentation products (acetate, ethanol, formate, xylooligosaccharides, and xylose) were analyzed by high-performance liquid chromatography using an Aminex HPX-87H column (Bio-Rad, Hercules, CA) at 60°C with a refractive index (RI) detector and 2.5 mM sulfuric acid as the mobile phase. Pellet nitrogen was measured in centrifuged pellet samples by using a TOC-V combustion analyzer coupled with a TNM-1 Total Nitrogen Module (Shimadzu Corporation, Columbia, MD) and comparing the results to a 1-g liter^−1^ glycine standard as previously described [7]. Concentrations of glucan, xylan, and arabinan in residual switchgrass samples were determined using quantitative saccharification from residual material at the end of incubation, as described in Sluiter *et al.* [23].

### Switchgrass glycomics

Glycome profiling analyses of switchgrass biomass residues were performed as described earlier [24,25], with increasingly harsh reagents (50 mM ammonium oxalate, 50 mM sodium carbonate, 1 M and 4 M potassium hydroxide, and sodium chlorite in glacial acetic acid) used for the serial fractionation of plant cell walls and the thorough detection of all epitopes present in the switchgrass biomass residues. The resulting cell wall extracts were probed by ELISA using a comprehensive suite of 155 monoclonal antibodies directed against all major plant cell wall glycans (see Additional file 1: Table S1). Cell wall glycan-directed monoclonal antibodies were procured as hybridoma cell culture supernatants stocks from Complex Carbohydrate Research Center, University of Georgia, and are available from CarboSource Services [(www.carbosource.net); JIM, MAC and CCRC series]. Additional antibodies were purchased from Biosupplies Australia, Parkville, Victoria, Australia (LAMP and BG-1 antibodies) and were used as per the manufacturer’s instructions. A detailed listing of all of the monoclonal antibodies used in this study is provided in Additional file 1: Table S1.

## Additional file

Additional file 1: Table S1. Listing of plant cell wall glycan-directed monoclonal antibodies (mAbs) used for glycome profiling analyses. The groupings of antibodies are based on a hierarchical clustering of ELISA data generated from a screen of all mAbs against a panel of plant polysaccharide preparations that groups the mAbs according to the predominant polysaccharides that they recognize. The majority of listings link to the Wall*Mab*DB plant cell wall monoclonal antibody database (http://www.wallmabdb.net) that provides detailed descriptions of each mAb, including immunogen, antibody isotype, epitope structure (to the extent known), supplier information, and related literature citations.

## Abbreviations

CBP: consolidated bioprocessing
MTC: Medium for thermophilic Clostridia
GH: glycoside hydrolase
