# Supplementary material for: Comparative analysis of the ability of Clostridium clariflavum strains and Clostridium thermocellum to utilize hemicellulose and unpretreated plant material
Source: Biotechnol Biofuels. 2014 Nov 18;7:136. doi: 10.1186/s13068-014-0136-4 (PMC4243297; doi:10.1186/s13068-014-0136-4)
Supplement: Additional file 1: Table S1. — Listing of plant cell wall glycan-directed monoclonal antibodies (mAbs) used for glycome profiling analyses. The groupings of antibodies are based on a hierarchical clustering of ELISA data generated from a screen of all mAbs against a panel of plant polysaccharide preparations that groups the mAbs according to the predominant polysaccharides that they recognize. The majority of listings link to the WallMabDB plant cell wall monoclonal antibody database (http://www.wallmabdb.net) that provides detailed descriptions of each mAb, including immunogen, antibody isotype, epitope structure (to the extent known), supplier information, and related literature citations. [file 13068_2014_136_MOESM1_ESM.docx]

**Additional file 1: Table S1**: Listing of plant cell wall glycan-directed monoclonal antibodies (mAbs) used for glycome profiling analyses. The groupings of antibodies are based on a hierarchical clustering of ELISA data generated from a screen of all mAbs against a panel of plant polysaccharide preparations^1,2^ that groups the mAbs according to the predominant polysaccharides that they recognize. The majority of listings link to the Wall*Mab*DB plant cell wall monoclonal antibody database (<http://www.wallmabdb.net>) that provides detailed descriptions of each mAb, including immunogen, antibody isotype, epitope structure (to the extent known), supplier information, and related literature citations.

**Glycan Group Recognized mAb Names**

| Non-Fucosylated Xyloglucan-1 | [CCRC-M95](http://glycomics.ccrc.uga.edu/wall2/jsp/abdetails.jsp?abnumber=162&abname=CCRC-M95) |
| --- | --- |
|  | [CCRC-M101](http://glycomics.ccrc.uga.edu/wall2/jsp/abdetails.jsp?abnumber=163&abname=CCRC-M101) |
|  |  |
| Non-Fucosylated Xyloglucan-2 | [CCRC-M104](http://glycomics.ccrc.uga.edu/wall2/jsp/abdetails.jsp?abnumber=164&abname=CCRC-M104) |
|  | [CCRC-M89](http://glycomics.ccrc.uga.edu/wall2/jsp/abdetails.jsp?abnumber=160&abname=CCRC-M89) |
|  | [CCRC-M93](http://glycomics.ccrc.uga.edu/wall2/jsp/abdetails.jsp?abnumber=161&abname=CCRC-M93) |
|  | [CCRC-M87](http://glycomics.ccrc.uga.edu/wall2/jsp/abdetails.jsp?abnumber=158&abname=CCRC-M87) |
|  | [CCRC-M88](http://glycomics.ccrc.uga.edu/wall2/jsp/abdetails.jsp?abnumber=159&abname=CCRC-M88) |
|  |  |
| Non-Fucosylated Xyloglucan-3 | [CCRC-M100](http://glycomics.ccrc.uga.edu/wall2/jsp/abdetails.jsp?abnumber=114&abname=CCRC-M100) |
|  | [CCRC-M103](http://glycomics.ccrc.uga.edu/wall2/jsp/abdetails.jsp?abnumber=113&abname=CCRC-M103) |
|  |  |
| Non-Fucosylated Xyloglucan-4 | [CCRC-M58](http://glycomics.ccrc.uga.edu/wall2/jsp/abdetails.jsp?abnumber=155&abname=CCRC-M58) |
|  | [CCRC-M86](http://glycomics.ccrc.uga.edu/wall2/jsp/abdetails.jsp?abnumber=157&abname=CCRC-M86) |
|  | [CCRC-M55](http://glycomics.ccrc.uga.edu/wall2/jsp/abdetails.jsp?abnumber=148&abname=CCRC-M55) |
|  | [CCRC-M52](http://glycomics.ccrc.uga.edu/wall2/jsp/abdetails.jsp?abnumber=145&abname=CCRC-M52) |
|  | [CCRC-M99](http://glycomics.ccrc.uga.edu/wall2/jsp/abdetails.jsp?abnumber=152&abname=CCRC-M99) |
|  |  |
| Non-Fucosylated Xyloglucan-5 | [CCRC-M54](http://glycomics.ccrc.uga.edu/wall2/jsp/abdetails.jsp?abnumber=147&abname=CCRC-M54) |
|  | [CCRC-M48](http://glycomics.ccrc.uga.edu/wall2/jsp/abdetails.jsp?abnumber=77&abname=CCRC-M48) |
|  | [CCRC-M49](http://glycomics.ccrc.uga.edu/wall2/jsp/abdetails.jsp?abnumber=76&abname=CCRC-M49) |
|  | [CCRC-M96](http://glycomics.ccrc.uga.edu/wall2/jsp/abdetails.jsp?abnumber=151&abname=CCRC-M96) |
|  | [CCRC-M50](http://glycomics.ccrc.uga.edu/wall2/jsp/abdetails.jsp?abnumber=143&abname=CCRC-M50) |
|  | [CCRC-M51](http://glycomics.ccrc.uga.edu/wall2/jsp/abdetails.jsp?abnumber=144&abname=CCRC-M51) |
|  | [CCRC-M53](http://glycomics.ccrc.uga.edu/wall2/jsp/abdetails.jsp?abnumber=146&abname=CCRC-M53) |
|  |  |
| Non-Fucosylated Xyloglucan-6 | [CCRC-M57](http://glycomics.ccrc.uga.edu/wall2/jsp/abdetails.jsp?abnumber=154&abname=CCRC-M57) |
|  |  |
| Fucosylated Xyloglucan | [CCRC-M102](http://glycomics.ccrc.uga.edu/wall2/jsp/abdetails.jsp?abnumber=142&abname=CCRC-M102) |
|  | [CCRC-M39](http://glycomics.ccrc.uga.edu/wall2/jsp/abdetails.jsp?abnumber=78&abname=CCRC-M39) |
|  | [CCRC-M106](http://glycomics.ccrc.uga.edu/wall2/jsp/abdetails.jsp?abnumber=112&abname=CCRC-M106) |
|  | [CCRC-M84](http://glycomics.ccrc.uga.edu/wall2/jsp/abdetails.jsp?abnumber=124&abname=CCRC-M84) |
|  | [CCRC-M1](http://glycomics.ccrc.uga.edu/wall2/jsp/abdetails.jsp?abnumber=1&abname=CCRC-M1) |
|  |  |
|  |  |
| Xylan-1/XG | [CCRC-M111](http://glycomics.ccrc.uga.edu/wall2/jsp/abdetails.jsp?abnumber=168&abname=CCRC-M111) |
|  | [CCRC-M108](http://glycomics.ccrc.uga.edu/wall2/jsp/abdetails.jsp?abnumber=149&abname=CCRC-M108) |
|  | [CCRC-M109](http://glycomics.ccrc.uga.edu/wall2/jsp/abdetails.jsp?abnumber=150&abname=CCRC-M109) |
|  |  |
| Xylan-2 | [CCRC-M119](http://glycomics.ccrc.uga.edu/wall2/jsp/abdetails.jsp?abnumber=106&abname=CCRC-M119) |
|  | [CCRC-M115](http://glycomics.ccrc.uga.edu/wall2/jsp/abdetails.jsp?abnumber=110&abname=CCRC-M115) |
|  | [CCRC-M110](http://glycomics.ccrc.uga.edu/wall2/jsp/abdetails.jsp?abnumber=167&abname=CCRC-M110) |
|  | [CCRC-M105](http://glycomics.ccrc.uga.edu/wall2/jsp/abdetails.jsp?abnumber=165&abname=CCRC-M105) |
|  |  |
| Xylan-3 | [CCRC-M117](http://glycomics.ccrc.uga.edu/wall2/jsp/abdetails.jsp?abnumber=108&abname=CCRC-M117) |
|  | [CCRC-M113](http://glycomics.ccrc.uga.edu/wall2/jsp/abdetails.jsp?abnumber=171&abname=CCRC-M113) |
|  | [CCRC-M120](http://glycomics.ccrc.uga.edu/wall2/jsp/abdetails.jsp?abnumber=105&abname=CCRC-M120) |
|  | [CCRC-M118](http://glycomics.ccrc.uga.edu/wall2/jsp/abdetails.jsp?abnumber=107&abname=CCRC-M118) |
|  | [CCRC-M116](http://glycomics.ccrc.uga.edu/wall2/jsp/abdetails.jsp?abnumber=109&abname=CCRC-M116) |
|  | [CCRC-M114](http://glycomics.ccrc.uga.edu/wall2/jsp/abdetails.jsp?abnumber=111&abname=CCRC-M114) |
|  |  |
| Xylan-4 | CCRC-M154 |
|  | CCRC-M150 |
|  |  |
| Xylan-5 | CCRC-M144 |
|  | CCRC-M146 |
|  | CCRC-M145 |
|  | CCRC-M155 |
|  |  |
| Xylan-6 | CCRC-M153 |
|  | CCRC-M151 |
|  | CCRC-M148 |
|  | CCRC-M140 |
|  | CCRC-M139 |
|  | CCRC-M138 |
|  |  |
| Xylan-7 | CCRC-M160 |
|  | [CCRC-M137](http://glycomics.ccrc.uga.edu/wall2/jsp/abdetails.jsp?abnumber=173&abname=CCRC-M137) |
|  | CCRC-M152 |
|  | CCRC-M149 |
|  |  |
| Galactomannan-1 | [CCRC-M75](http://glycomics.ccrc.uga.edu/wall2/jsp/abdetails.jsp?abnumber=133&abname=CCRC-M75) |
|  | [CCRC-M70](http://glycomics.ccrc.uga.edu/wall2/jsp/abdetails.jsp?abnumber=61&abname=CCRC-M70) |
|  | [CCRC-M74](http://glycomics.ccrc.uga.edu/wall2/jsp/abdetails.jsp?abnumber=134&abname=CCRC-M74) |
|  |  |
| Galactomannan-2 | CCRC-M166 |
|  | CCRC-M168 |
|  | CCRC-M174 |
|  | CCRC-M175 |
| Acetylated Mannan | CCRC-M169 |
|  | CCRC-M170 |
|  |  |
| β-Glucan | [LAMP](http://glycomics.ccrc.uga.edu/wall2/jsp/abdetails.jsp?abnumber=47&abname=LAMP2H12H7) |
|  | [BG1](http://glycomics.ccrc.uga.edu/wall2/jsp/abdetails.jsp?abnumber=48&abname=BG1) |
|  |  |
| HG Backbone-1 | [CCRC-M131](http://glycomics.ccrc.uga.edu/wall2/jsp/abdetails.jsp?abnumber=181&abname=CCRC-M131) |
|  | [CCRC-M38](http://glycomics.ccrc.uga.edu/wall2/jsp/abdetails.jsp?abnumber=45&abname=CCRC-M38) |
|  | [JIM5](http://glycomics.ccrc.uga.edu/wall2/jsp/abdetails.jsp?abnumber=14&abname=JIM5) |
|  |  |
| HG Backbone-2 | [JIM136](http://glycomics.ccrc.uga.edu/wall2/jsp/abdetails.jsp?abnumber=57&abname=JIM136) |
|  | [JIM7](http://glycomics.ccrc.uga.edu/wall2/jsp/abdetails.jsp?abnumber=13&abname=JIM7) |
|  |  |
| RG-I Backbone | [CCRC-M69](http://glycomics.ccrc.uga.edu/wall2/jsp/abdetails.jsp?abnumber=172&abname=CCRC-M69) |
|  | [CCRC-M35](http://glycomics.ccrc.uga.edu/wall2/jsp/abdetails.jsp?abnumber=66&abname=CCRC-M35) |
|  | [CCRC-M36](http://glycomics.ccrc.uga.edu/wall2/jsp/abdetails.jsp?abnumber=37&abname=CCRC-M36) |
|  | [CCRC-M14](http://glycomics.ccrc.uga.edu/wall2/jsp/abdetails.jsp?abnumber=67&abname=CCRC-M14) |
|  | [CCRC-M129](http://glycomics.ccrc.uga.edu/wall2/jsp/abdetails.jsp?abnumber=104&abname=CCRC-M129) |
|  | [CCRC-M72](http://glycomics.ccrc.uga.edu/wall2/jsp/abdetails.jsp?abnumber=135&abname=CCRC-M72) |
|  |  |
| Linseed Mucilage RG-I | [JIM3](http://glycomics.ccrc.uga.edu/wall2/jsp/abdetails.jsp?abnumber=79&abname=JIM1) |
|  | [CCRC-M40](http://glycomics.ccrc.uga.edu/wall2/jsp/abdetails.jsp?abnumber=83&abname=CCRC-M40) |
|  | CCRC-M161 |
|  | CCRC-M164 |
|  |  |
| Physcomitrella Pectin | [CCRC-M98](http://glycomics.ccrc.uga.edu/wall2/jsp/abdetails.jsp?abnumber=115&abname=CCRC-M98) |
|  | [CCRC-M94](http://glycomics.ccrc.uga.edu/wall2/jsp/abdetails.jsp?abnumber=118&abname=CCRC-M94) |
|  |  |
| RG-Ia | [CCRC-M5](http://glycomics.ccrc.uga.edu/wall2/jsp/abdetails.jsp?abnumber=81&abname=CCRC-M5) |
|  | [CCRC-M2](http://glycomics.ccrc.uga.edu/wall2/jsp/abdetails.jsp?abnumber=8&abname=CCRC-M2) |
|  |  |
| RG-Ib | [JIM137](http://glycomics.ccrc.uga.edu/wall2/jsp/abdetails.jsp?abnumber=58&abname=JIM137) |
|  | [JIM101](http://glycomics.ccrc.uga.edu/wall2/jsp/abdetails.jsp?abnumber=55&abname=JIM101) |
|  | [CCRC-M61](http://glycomics.ccrc.uga.edu/wall2/jsp/abdetails.jsp?abnumber=138&abname=CCRC-M61) |
|  | [CCRC-M30](http://glycomics.ccrc.uga.edu/wall2/jsp/abdetails.jsp?abnumber=33&abname=CCRC-M30) |
|  |  |
| RG-Ic | [CCRC-M23](http://glycomics.ccrc.uga.edu/wall2/jsp/abdetails.jsp?abnumber=92&abname=CCRC-M23) |
|  | [CCRC-M17](http://glycomics.ccrc.uga.edu/wall2/jsp/abdetails.jsp?abnumber=74&abname=CCRC-M17) |
|  | [CCRC-M19](http://glycomics.ccrc.uga.edu/wall2/jsp/abdetails.jsp?abnumber=0&abname=CCRC-M19) |
|  | [CCRC-M18](http://glycomics.ccrc.uga.edu/wall2/jsp/abdetails.jsp?abnumber=0&abname=CCRC-M18) |
|  | [CCRC-M56](http://glycomics.ccrc.uga.edu/wall2/jsp/abdetails.jsp?abnumber=141&abname=CCRC-M56) |
|  | [CCRC-M16](http://glycomics.ccrc.uga.edu/wall2/jsp/abdetails.jsp?abnumber=73&abname=CCRC-M16) |
|  |  |
|  |  |
| RG-I/Arabinogalactan | [CCRC-M60](http://glycomics.ccrc.uga.edu/wall2/jsp/abdetails.jsp?abnumber=139&abname=CCRC-M60) |
|  | [CCRC-M41](http://glycomics.ccrc.uga.edu/wall2/jsp/abdetails.jsp?abnumber=82&abname=CCRC-M41) |
|  | [CCRC-M80](http://glycomics.ccrc.uga.edu/wall2/jsp/abdetails.jsp?abnumber=128&abname=CCRC-M80) |
|  | [CCRC-M79](http://glycomics.ccrc.uga.edu/wall2/jsp/abdetails.jsp?abnumber=129&abname=CCRC-M79) |
|  | [CCRC-M44](http://glycomics.ccrc.uga.edu/wall2/jsp/abdetails.jsp?abnumber=68&abname=CCRC-M44) |
|  | [CCRC-M33](http://glycomics.ccrc.uga.edu/wall2/jsp/abdetails.jsp?abnumber=75&abname=CCRC-M33) |
|  | [CCRC-M32](http://glycomics.ccrc.uga.edu/wall2/jsp/abdetails.jsp?abnumber=35&abname=CCRC-M32) |
|  | [CCRC-M13](http://glycomics.ccrc.uga.edu/wall2/jsp/abdetails.jsp?abnumber=43&abname=CCRC-M13) |
|  | [CCRC-M42](http://glycomics.ccrc.uga.edu/wall2/jsp/abdetails.jsp?abnumber=86&abname=CCRC-M42) |
|  | [CCRC-M24](http://glycomics.ccrc.uga.edu/wall2/jsp/abdetails.jsp?abnumber=93&abname=CCRC-M24) |
|  | [CCRC-M12](http://glycomics.ccrc.uga.edu/wall2/jsp/abdetails.jsp?abnumber=71&abname=CCRC-M12) |
|  | [CCRC-M7](http://glycomics.ccrc.uga.edu/wall2/jsp/abdetails.jsp?abnumber=3&abname=CCRC-M7) |
|  | [CCRC-M77](http://glycomics.ccrc.uga.edu/wall2/jsp/abdetails.jsp?abnumber=131&abname=CCRC-M77) |
|  | [CCRC-M25](http://glycomics.ccrc.uga.edu/wall2/jsp/abdetails.jsp?abnumber=84&abname=CCRC-M25) |
|  | [CCRC-M9](http://glycomics.ccrc.uga.edu/wall2/jsp/abdetails.jsp?abnumber=69&abname=CCRC-M9) |
|  | [CCRC-M128](http://glycomics.ccrc.uga.edu/wall2/jsp/abdetails.jsp?abnumber=183&abname=CCRC-M128) |
|  | [CCRC-M126](http://glycomics.ccrc.uga.edu/wall2/jsp/abdetails.jsp?abnumber=184&abname=CCRC-M126) |
|  | [CCRC-M134](http://glycomics.ccrc.uga.edu/wall2/jsp/abdetails.jsp?abnumber=102&abname=CCRC-M134) |
|  | [CCRC-M125](http://glycomics.ccrc.uga.edu/wall2/jsp/abdetails.jsp?abnumber=185&abname=CCRC-M125) |
|  | [CCRC-M123](http://glycomics.ccrc.uga.edu/wall2/jsp/abdetails.jsp?abnumber=187&abname=CCRC-M123) |
|  | [CCRC-M122](http://glycomics.ccrc.uga.edu/wall2/jsp/abdetails.jsp?abnumber=188&abname=CCRC-M122) |
|  | [CCRC-M121](http://glycomics.ccrc.uga.edu/wall2/jsp/abdetails.jsp?abnumber=189&abname=CCRC-M121) |
|  | [CCRC-M112](http://glycomics.ccrc.uga.edu/wall2/jsp/abdetails.jsp?abnumber=169&abname=CCRC-M112) |
|  | [CCRC-M21](http://glycomics.ccrc.uga.edu/wall2/jsp/abdetails.jsp?abnumber=88&abname=CCRC-M21) |
|  | [JIM131](http://glycomics.ccrc.uga.edu/wall2/jsp/abdetails.jsp?abnumber=94&abname=JIM131) |
|  | [CCRC-M22](http://glycomics.ccrc.uga.edu/wall2/jsp/abdetails.jsp?abnumber=46&abname=CCRC-M22) |
|  | [JIM132](http://glycomics.ccrc.uga.edu/wall2/jsp/abdetails.jsp?abnumber=56&abname=JIM132) |
|  | [JIM1](http://glycomics.ccrc.uga.edu/wall2/jsp/abdetails.jsp?abnumber=79&abname=JIM1) |
|  | [CCRC-M15](http://glycomics.ccrc.uga.edu/wall2/jsp/abdetails.jsp?abnumber=72&abname=CCRC-M15) |
|  | [CCRC-M8](http://glycomics.ccrc.uga.edu/wall2/jsp/abdetails.jsp?abnumber=29&abname=CCRC-M8) |
|  | [JIM16](http://glycomics.ccrc.uga.edu/wall2/jsp/abdetails.jsp?abnumber=62&abname=JIM16) |
|  |  |
| Arabinogalactan-1 | [JIM93](http://glycomics.ccrc.uga.edu/wall2/jsp/abdetails.jsp?abnumber=117&abname=JIM93) |
|  | [JIM94](http://glycomics.ccrc.uga.edu/wall2/jsp/abdetails.jsp?abnumber=95&abname=JIM94) |
|  | [JIM11](http://glycomics.ccrc.uga.edu/wall2/jsp/abdetails.jsp?abnumber=41&abname=JIM11) |
|  | [MAC204](http://glycomics.ccrc.uga.edu/wall2/jsp/abdetails.jsp?abnumber=23&abname=MAC204) |
|  | [JIM20](http://glycomics.ccrc.uga.edu/wall2/jsp/abdetails.jsp?abnumber=91&abname=JIM20) |
|  |  |
| Arabinogalactan-2 | [JIM14](http://glycomics.ccrc.uga.edu/wall2/jsp/abdetails.jsp?abnumber=31&abname=JIM14) |
|  | [JIM19](http://glycomics.ccrc.uga.edu/wall2/jsp/abdetails.jsp?abnumber=44&abname=JIM19) |
|  | [JIM12](http://glycomics.ccrc.uga.edu/wall2/jsp/abdetails.jsp?abnumber=191&abname=JIM12) |
|  | [CCRC-M133](http://glycomics.ccrc.uga.edu/wall2/jsp/abdetails.jsp?abnumber=96&abname=JIM133) |
|  | [CCRC-M107](http://glycomics.ccrc.uga.edu/wall2/jsp/abdetails.jsp?abnumber=166&abname=CCRC-M107) |
|  |  |
| Arabinogalactan-3 | [JIM4](http://glycomics.ccrc.uga.edu/wall2/jsp/abdetails.jsp?abnumber=40&abname=JIM4) |
|  | [CCRC-M31](http://glycomics.ccrc.uga.edu/wall2/jsp/abdetails.jsp?abnumber=34&abname=CCRC-M31) |
|  | [JIM17](http://glycomics.ccrc.uga.edu/wall2/jsp/abdetails.jsp?abnumber=39&abname=JIM17) |
|  | [CCRC-M26](http://glycomics.ccrc.uga.edu/wall2/jsp/abdetails.jsp?abnumber=85&abname=CCRC-M26) |
|  | [JIM15](http://glycomics.ccrc.uga.edu/wall2/jsp/abdetails.jsp?abnumber=32&abname=JIM15) |
|  | [JIM8](http://glycomics.ccrc.uga.edu/wall2/jsp/abdetails.jsp?abnumber=80&abname=JIM8) |
|  | [CCRC-M85](http://glycomics.ccrc.uga.edu/wall2/jsp/abdetails.jsp?abnumber=121&abname=CCRC-M85) |
|  | [CCRC-M81](http://glycomics.ccrc.uga.edu/wall2/jsp/abdetails.jsp?abnumber=127&abname=CCRC-M81) |
|  | [MAC266](http://glycomics.ccrc.uga.edu/wall2/jsp/abdetails.jsp?abnumber=98&abname=MAC266) |
|  | [PN 16.4B4](http://glycomics.ccrc.uga.edu/wall2/jsp/abdetails.jsp?abnumber=11&abname=PN%2016.4B4) |
|  |  |
| Arabinogalactan-4 | [MAC207](http://glycomics.ccrc.uga.edu/wall2/jsp/abdetails.jsp?abnumber=22&abname=MAC207) |
|  | [JIM133](http://glycomics.ccrc.uga.edu/wall2/jsp/abdetails.jsp?abnumber=96&abname=JIM133) |
|  | [JIM13](http://glycomics.ccrc.uga.edu/wall2/jsp/abdetails.jsp?abnumber=30&abname=JIM13) |
|  | [CCRC-M92](http://glycomics.ccrc.uga.edu/wall2/jsp/abdetails.jsp?abnumber=119&abname=CCRC-M92) |
|  | [CCRC-M91](http://glycomics.ccrc.uga.edu/wall2/jsp/abdetails.jsp?abnumber=120&abname=CCRC-M91) |
|  | [CCRC-M78](http://glycomics.ccrc.uga.edu/wall2/jsp/abdetails.jsp?abnumber=130&abname=CCRC-M78) |
|  |  |
| Unidentified | [MAC265](http://glycomics.ccrc.uga.edu/wall2/jsp/abdetails.jsp?abnumber=97&abname=MAC265) |
|  | [CCRC-M97](http://glycomics.ccrc.uga.edu/wall2/jsp/abdetails.jsp?abnumber=116&abname=CCRC-M97) |
|  |  |

References:

1. Pattathil S, Avci U, Baldwin D et al. A comprehensive toolkit of plant cell wall glycan-directed monoclonal antibodies. *Plant Physiol* 2010; 153:514-525.

2. Pattathil S, Avci U, Hahn MG Immunological approaches to plant cell wall and biomass characterization: glycome profiling. *Methods Mol Biol* 2012, 908: 61-72.
